# The Long-Term Care Financing Challenge: What Role for Home Equity?

**DOI:** 10.1093/geroni/igaa057.2418

**Published:** 2020-12-16

**Authors:** Jane Tavares, Marc Cohen, Lauren Popham

**Affiliations:** 1 University of Massachusetts Boston, Florence, Massachusetts, United States; 2 University of Massachusetts Boston, Boston, Massachusetts, United States; 3 National Council on Aging, Arlington, Virginia, United States

## Abstract

With increasing longevity comes the need for support in activities of daily living. This growing demand for long-term services and supports (LTSS) threatens older adult financial security. About 25 million adults aged 65 and older will have a significant LTSS need that will cost up to $140,000 out-of-pocket plus an estimated $120,000 from public payers like Medicaid; the total public and private costs of care exceeding $250,000. Home equity, the most valuable financial asset owned by Americans, offers one solution to this financing challenge. While seniors boast $7 trillion in collective home equity, opportunities to annuitize this asset vary and the distributions of home ownership and value are highly skewed. Using the 2016 wave of the HRS, this paper explores the relationship between socio-demographic/health characteristics and the annuitized home value. We determine which individuals with LTSS needs might benefit from the annuitization of home equity and to what extent.

